# Effective Use of Water in Crop Plants in Dryland Agriculture: Implications of Reactive Oxygen Species and Antioxidative System

**DOI:** 10.3389/fpls.2021.778270

**Published:** 2022-01-10

**Authors:** Jagadish Rane, Ajay Kumar Singh, Manish Tiwari, P. V. Vara Prasad, S. V. Krishna Jagadish

**Affiliations:** ^1^ICAR-National Institute of Abiotic Stress Management, Baramati, India; ^2^Department of Agronomy, Kansas State University, Manhattan, KS, United States

**Keywords:** water productivity, antioxidant system, reactive oxygen species, crop plants, drought tolerance

## Abstract

Under dryland conditions, annual and perennial food crops are exposed to dry spells, severely affecting crop productivity by limiting available soil moisture at critical and sensitive growth stages. Climate variability continues to be the primary cause of uncertainty, often making timing rather than quantity of precipitation the foremost concern. Therefore, mitigation and management of stress experienced by plants due to limited soil moisture are crucial for sustaining crop productivity under current and future harsher environments. Hence, the information generated so far through multiple investigations on mechanisms inducing drought tolerance in plants needs to be translated into tools and techniques for stress management. Scope to accomplish this exists in the inherent capacity of plants to manage stress at the cellular level through various mechanisms. One of the most extensively studied but not conclusive physiological phenomena is the balance between reactive oxygen species (ROS) production and scavenging them through an antioxidative system (AOS), which determines a wide range of damage to the cell, organ, and the plant. In this context, this review aims to examine the possible roles of the ROS-AOS balance in enhancing the effective use of water (EUW) by crops under water-limited dryland conditions. We refer to EUW as biomass produced by plants with available water under soil moisture stress rather than per unit of water (WUE). We hypothesize that EUW can be enhanced by an appropriate balance between water-saving and growth promotion at the whole-plant level during stress and post-stress recovery periods. The ROS-AOS interactions play a crucial role in water-saving mechanisms and biomass accumulation, resulting from growth processes that include cell division, cell expansion, photosynthesis, and translocation of assimilates. Hence, appropriate strategies for manipulating these processes through genetic improvement and/or application of exogenous compounds can provide practical solutions for improving EUW through the optimized ROS-AOS balance under water-limited dryland conditions. This review deals with the role of ROS-AOS in two major EUW determining processes, namely water use and plant growth. It describes implications of the ROS level or content, ROS-producing, and ROS-scavenging enzymes based on plant water status, which ultimately affects photosynthetic efficiency and growth of plants.

## Introduction

Drought is a challenging natural phenomenon frequently occurring in arid, semiarid, and sub-humid dryland environments. However, agricultural drought is typically defined in terms of soil moisture deficit relative to the needs of crops at particular growth and developmental stages. Drought stress for plants results from insufficient amount of water available for the maintenance of normal physiological processes, such as photosynthesis, respiration, and cell, tissue, organ, and plant homeostasis (Lawas et al., 2019a,b). By adversely affecting vital physiological processes, drought leads to a substantial reduction in the productivity of crops. With the predicted increase in precipitation variability across the globe, the intensity and duration of extreme drought stress are likely to increase, adding more pressure on agri-food systems to meet the increasing demand by a growing population. In countries like India and sub-Saharan Africa, major proportion of the crop production area is drought prone, and drought stress can lead to significant productivity losses in annual and perennial crops (Hyland and Russ, 2019; Choudhury et al., 2021). Nearly 68% of total arable land in India is prone to drought stress due to erratic and irregular rainfall (Das et al., 2020). Similarly, in Africa, about 70% of arable land includes 66% of the cereal-producing area, and 80% of livestock holdings, which are under dryland conditions and prone to drought stress (Morris et al., 2015; Halilou et al., 2020). Not only in underdeveloped or developing countries but also substantial arable areas in developed countries, including the United States (Stewart et al., 2006), Australia (Howden et al., 2014), and Mediterranean regions (Ryan et al., 2006) are prone to drought stress. Recently, a modeling study by Williams et al. (2020) has shown that the period from 2000 to 2018 was the driest phase since the late 1500s, and, consequently, many regions in the United States may be on the verge of entering a mega drought period. Severe drought has already been experienced in western parts of the United States in the last few years. Model projections show robust drying and increases in extreme drought in many regions around the world by the end of the 21st century, including regions in America, Europe, Asia, and Australia (Cook et al., 2020). Hence, sustaining the productivity of crops under drought stress is crucial for income-generating and sustaining livelihoods of farmers across developing and developed countries. Increasing demand for food, with limited resources and predicted adverse effects of climate change, has emphasized the need for sustaining productivity under dryland agriculture. Hence, scientific interventions are necessary to develop and produce genetically improved drought-tolerant crop varieties (Pareek et al., 2020). To achieve targeted levels of drought tolerance, it is necessary to revisit and decipher the mechanisms associated with survival, tolerance, and recovery of drought-affected plants at molecular, cellular, and at the whole plant levels.

To survive under water-limited conditions, crop plants have evolved three different mechanisms, namely escape, avoidance, and tolerance (Kaya and Zeki, 2017; Blumenthal et al., 2020). One of the immediate responses of crops to drought is the partial closure of stomata, which can reduce water loss through transpiration and limit the entry of CO_2_ (Ranjbar et al., 2021). Apart from morphological changes in response to drought, several intrinsic molecular mechanisms are activated upon stress signaling and perception. One of the well-established stress responses includes the generation of reactive oxygen species (ROS), such as hydrogen peroxides (H_2_O_2_), superoxide (O_2_^–^), hydroxyl radical (OH^–^), and singlet oxygen (^1^O_2_) (Srivastava and Suprassana, 2015). ROS production is directly related to stress in plants and leads to membrane damage and subsequent stress signaling (Mishra et al., 2017, 2018). ROS production takes place in the reaction centers of photosystem I (PSI) and PSII in chloroplast thylakoids (Foyer, 2018; Mishra et al., 2021a). ROS species formation is a result of the direct transfer of the excitation energy from chlorophyll to produce singlet oxygen, or by oxygen reduction in the Mehler reaction (Foyer, 2018). In addition to the chloroplast, ROS are generated in mitochondria (Moller et al., 2007), peroxisomes (del Río and López-Huertas, 2016), and even in intercellular space of the cells, the apoplast (Moschou et al., 2008; Miller et al., 2009). Antioxidant system (AOS) is the “first line of defense” against ROS generated by environmental stimuli (Srivastava and Suprassana, 2015). The ROS species are interchangeably produced by redox reactions involving several enzymes. The univalent reduction of O_2_^–^ in the presence of enzyme superoxide dismutase (SOD) produces H_2_O_2_, and a molecule of oxygen or H_2_O_2_ in the presence of Fe^2+^ gives rise to hydroxyl radical (Fukai and Ushio-Fukai, 2011). H_2_O_2_ inactivates most of the enzymes by oxidizing their thiol groups, and, because of its long-life span compared to other ROS (half-life of milliseconds to seconds) (Marinho et al., 2014) and high permeability across membranes, H_2_O_2_ is considered as a secondary messenger at low concentrations; however, at high concentrations, it causes programmed cell death (Quan et al., 2008; Zheng et al., 2020). H_2_O_2_ metabolism and signaling cascades play important roles during plant growth and development, such as xylem differentiation, development of root hair, reinforcement of a plant cell wall by structural cross-linking, and loosening, leading to stomatal control (Saxena et al., 2016). Similar to H_2_O_2_, OH^–^ free radical can interact with all biological macromolecules and play a signaling role during plant development. The oxidative power of OH^–^ is utilized during vegetative growth, reproduction, and adaptation to stress by stomatal closure (Richards et al., 2015). Due to the lack of an enzymatic mechanism, it is hard to eliminate this highly reactive radical, and its enhanced accumulation leads to cell death (Das and Roychoudhury, 2014). The enzymes, namely catalase (CAT), and different types of peroxidases can scavenge H_2_O_2_ from the cell (Mishra et al., 2021a). Similar to the enzymatic antioxidant defense system, the plant also possesses non-enzymatic antioxidants like carotenoids, flavonoids, and ascorbate, which are synthesized in response to excess light stress and complement the antioxidant enzymes in mitigating ROS (Hatier and Gould, 2008; Agati et al., 2009, 2011). Compounds such as phenylpropanoids have tremendous potential to reduce ROS concentration and are synthesized in response to different abiotic stresses, including drought (Agati and Tattini, 2010; Pollastri and Tattini, 2011; Ding et al., 2021).

It is evident from previous reviews that a lot of knowledge has been generated on ROS-AOS in the context of different stresses, including drought (Henmi et al., 2007; Claeys and Inze, 2013; Song et al., 2014; You and Chan, 2015; Ullah et al., 2017; Devireddy et al., 2020; Hasanuzzaman et al., 2020). In addition, various cellular processes that lead to the generation of ROS and scavenging or detoxification of these deleterious hyper reactive molecules and their relevance to perception of drought stress in crops have been reviewed (Noctor et al., 2014; Verma et al., 2019).

This review complements previous reports and covers the ROS-AOS system in facilitating effective water use (EUW). Furthermore, it provides an optimistic insight into scientific investigations, particularly on critical cellular mechanisms, which can be translated into tools or products for alleviating oxidative stress. We refer to EUW as biomass produced by plants with available water under soil moisture stress rather than per unit of water (WUE). Blum (2009) introduced that EUW implies maximum utilization of soil moisture through transpiration by minimizing direct water loss by soil evaporation or other non-stomatal mechanisms. Thus, EUW-associated traits promise to allow judicious use of the available soil moisture by plant and retaining moisture during critical and sensitive crop growth and development stages. While mining for drought tolerance traits in crops is in progress, considering EUW to complement WUE has been recommended for increased genetic gains and enhanced crop productivity under drought stress (Blum, 2009). Emphasis on EUW is based on the argument that many traits that contribute to WUE come at the cost of plants performance under favorable conditions. It is well-known that stomatal mechanisms that determine water use are influenced by ROS (Noctor et al., 2014). On the other hand, cell division and cell expansion, in addition to photosynthesis, the primary processes of biomass accumulation, are also affected. In this context, we discuss the role of ROS-AOS in two major EUW-determining processes, namely stomatal regulation of water use and plant growth. This review aims to analyze information and knowledge related to ROS-AOS in plants for facilitating EUW, particularly for drought stress management in crops.

## Influence of Reactive Oxygen Species-Antioxidative System on Plant Processes

### Plant Water Relations

Water stress severely impacts routine plant functionality and triggers molecular, biochemical, morphological, and physiological mechanisms to compensate for water limitation (Mitchell et al., 2013; Lee et al., 2016). Water limitation causes a surge in the production of ROS species such as H_2_O_2_, and superoxide anion radicals (O_2_^–^) resulting in lipid peroxidation, reduced photosynthetic capacity (Deeba et al., 2012), enhanced programmed cell death (Gill and Tuteja, 2010), and retarded growth (Wallace et al., 2016). The ROS-scavenging antioxidant enzymes, such as SOD, POD, CAT, glutathione reductase (GR), and ascorbate peroxidase (APX), can be triggered to neutralize excessive ROS accumulation. Alterations in these enzyme activities are the primary pathway in plants for inducing drought stress tolerance (Gill and Tuteja, 2010; Nikoleta-Kleio et al., 2020). ROS-AOS can act through root as well as shoot components, associated with water uptake and transpiration (Zhang et al., 2019). Besides, root and shoot, one of the well-characterized physiological responses under drought stress is ABA-induced leaf stomatal closure (Cruz de Carvalho, 2008). An insight into the diurnal and seasonal implication of such action by ROS-AOS can help us tailor management options to optimize water use by plants. Hence, this section discusses the role of ROS-AOS in processes regulating water relations.

It is well established that water relations at the shoot level are primarily determined by stomatal mechanisms that regulate transpiration. ROS can influence guard cell functions in leaves’ in plants, including *Arabidopsis*, bean (*Phaseolus* sp.), and pea (*Pisum* sp.) (Song et al., 2014). Most of the ROS-related actions appear to be associated with abscisic acid (ABA). The phytohormone ABA is synthesized in shoots, roots, particularly in seeds, and leaves (veins, and guard cells) (Boursiac et al., 2013), and plays a critical role in stomatal regulation in response to abiotic stresses. ABA actions involve methyl jasmonate (MeJA), which evokes ROS and NO (nitrous oxide) production in guard cells as shown in *A. thaliana* (Islam et al., 2009; Nazareno and Hernandez, 2017). Zhu et al. (2014) found that disruption of partially redundant Nicotinamide Adenine Dinucleotide Phosphate-reduced form (NADPH) oxidase catalytic subunit genes expressed in guard cells stops Methyl Jasmonate (MeJA)-induced stomatal closure and ROS production. This indicated that the two NADPH oxidases are chief ROS sources in guard cell MeJA signaling. However, it does not cause MeJA-induced cytosolic alkalization in *Arabidopsis* guard cells, suggesting the possible role of MeJA-induced ROS and NO in stomatal closure and drought tolerance (Ye et al., 2013; Nazareno and Hernandez, 2017). Another NADPH oxidase-interacting gene, *OPEN STOMATA 1* (*OST1*), acts upstream of ROS signaling to mediate ABA-induced stomatal closure (Acharya et al., 2013; Ali et al., 2019). The *OST1* regulates ROS generation through direct interaction and phosphorylation of the NADPH oxidase subunit of RBOHF (respiratory burst oxidase homologs) in *Arabidopsis* (Sirichandra et al., 2009; Han et al., 2019). MeJA also elicits NO production in the guard cells for induction of stomatal closure through the release of Ca^2+^ (Han et al., 2019). Besides NADPH oxidases, other ROS-producing enzymes (cell wall bound-peroxidase and copper amine oxidase) have been shown to regulate stomatal movement (Khokon et al., 2011). Several genes have been characterized, which reveal the role of ROS species in stomatal closure under stress conditions. The RING finger ubiquitin E3 ligase, OsHTAS, can promote H_2_O_2_-induced stomatal closure, leading to ABA-dependent drought, heat, and salt tolerance (Liu et al., 2016). *Arabidopsis* CYCLIN H;1 (CYCH;1) positively regulates blue light-induced stomatal opening by controlling ROS homeostasis (Zhou et al., 2014). Another critical interaction involved in stomatal closure during drought stress includes biological gas transmitter H_2_S (hydrogen sulfide), which promotes *de novo* ABA synthesis and activation of AOS to provide drought stress tolerance in *Arabidopsis* and rice (Jin et al., 2013; Zhou et al., 2020).

Water uptake and transport are the crucial processes determining the use of water by plants. Osmotic and hydrostatic forces are the major determinants of water uptake through the root. Osmotic force results from accumulation of solutes, and, furthermore, addition of root pressure and hydrostatic force produced mainly by transpiration stream drives movement of water (Kim et al., 2018). Adverse effect of ROS on functions of roots, the major component of plant water uptake structure, can negatively influence the water relations in plants (An et al., 2018). Under different abiotic stress conditions, root tissue produces ROS, and, among them, the role of H_2_O_2_ in root water uptake has been extensively investigated (Boursiac et al., 2008). It is shown that exogenous application of H_2_O_2_ inhibited cell and root hydraulic conductivity by closing aquaporin pores (Ye and Steudle, 2006) or accumulation of plasma membrane intrinsic proteins (PIPs) in internal structures called vesicles and vacuoles (Boursiac et al., 2008). Lack of function of genes associated with oxidative burst in the root can lead to accumulation of ROS in root vascular system and the xylem vessel of *Arabidopsis* subjected to salinity that creates osmotic stress similar to the one caused by soil moisture stress (Jiang et al., 2012). Production of ROS in maize (*Zea mays*) and *Arabidopsis* roots is associated with wall loosening and growth *via* cell elongation (Mangano et al., 2017). RAC/ROP-regulated production of NADPH-dependent H_2_O_2_ has been implicated in the secondary cell wall differentiation (Oh et al., 2018). Drought stress tolerance can be achieved by expressing genes involved in enhanced root growth and minimizing ROS levels. In this context, a multiple stress-responsive *WRKY* gene, *GmWRKY27*, interacts with *GmMYB174* to suppress *GmNAC29* expression, resulting in reduced ROS levels. The transgenic hairy roots of soybean (*Glycine max*) overexpressing *GmWRKY27* displayed better root growth and enhanced tolerance to drought and salt stress than control plants (Wang et al., 2015). ROS can act as signals to regulate root hydraulic conductivity under stress conditions and increase or decrease root hydraulic conductivity, depending on the cellular concentration of ROS (Kaneko et al., 2015). This hypothesis is supported by the observations that applying exogenous antioxidants can modify ROS levels and the response of ABA on root hydraulic conductivity (Xu et al., 2015). Additionally, ROS-triggered intracellular trafficking of aquaporin negatively impacts water transport (Boursiac et al., 2008; Quiroga et al., 2018). The effect is caused by direct ROS-mediated closure or by more indirect signaling pathways, and the latter possibly involves salicylic acid (Ye and Steudle, 2006; Boursiac et al., 2008; Quiroga et al., 2018). Apart from ROS-mediated regulation of aquaporins, water transport can also be influenced by the activity of ROS in other pathways involving genes implicated in the regulation of root growth and development under drought stress (Janiak et al., 2015; Dalal et al., 2018). Furthermore, exogenous application of putrescine (Put) exhibited reduced water loss by transpiration and increased drought tolerance. The effect can be attributed to antioxidant or ROS scavenging action in roots and activation of stress-related genes (Alcazar et al., 2010; Mahdavian et al., 2021). Putrescine is also known to alleviate the inhibitory action of aluminum-induced ROS and provide tolerance against excess aluminum and heat stress (Fini et al., 2012; Yu et al., 2019; Kolupaev et al., 2021). Proper functioning of stomata requires coordination between ABA, ROS, and Ca^2+^-based signaling systems. Alteration in this may disturb the gaseous balance and plant water status, which ultimately affects the photosynthetic efficiency and growth of plants (Srivastava and Suprassana, 2015).

In summary, the ROS-AOS system under water-limited conditions functionally regulates the growth and development of plant organs, including roots and shoots. The stomatal closure in leaves is a crucial process that provides drought tolerance to plants. ROS-mediated actions involving a key player, ABA in its nexus, govern stomatal movements during stress. Not only ABA but another phytohormone MeJA is recruited by ABA to induce ROS machinery and NO production in guard cells, resulting in stomatal closure during drought conditions. NADPH oxidases work under the ABA-MeJA nexus and upstream of ROS system to critically elevate ROS levels under drought. In addition to the effect on aboveground parts, i.e., leaves, ROS-AOS system defines root water uptake and transport functionality under water stress. Interestingly, a large knowledge gap exists in determining the role of ABA-MeJA-NADPH-oxidase circuitry in root tissue to regulate ROS levels. Future studies targeting this regulatory network in root tissue may lead to better water uptake and transport under water-limited conditions. Such regulations, if fine-tuned to extend the availability of water, can extend the life of crop plants till the subsequent spell of water supply either through natural precipitation or life-saving irrigation and, hence, can substantially improve EUW, particularly in the events of mid-season drought.

### Plant Growth

When subjected to soil moisture stress, generally, plants cease their growth, and much of the plant processes and assimilates are diverted for survival or for ensuring a successful reproductive cycle (Mi et al., 2018; Chadha et al., 2019). This strategy allows plants to save water by reducing the size of leaves associated with transpiration or curtailing the duration of growth to escape severe stress (Mi et al., 2018). Annual crops are featured by distinct growth phases for vegetative and reproductive stages, often separated by the time of flowering, which is highly critical to adapting to the prevailing environment (Craufurd and Wheeler, 2009). On the other hand, perennials, including forage grasses and fruit crops, have different growing habits where both vegetative and reproductive parts grow simultaneously (Skinner and Moore, 2007). Hence, the time of occurrence of soil moisture stress determines the differences in responses and impacts on biomass production and productivity among these crops (Lelievre et al., 2011). Plant growth, reflected by an increase in biomass, results from a set of primary processes, which include cell division and expansion, assimilate accumulation through photosynthesis, and transport of these assimilates to growing parts (Gonzalez et al., 2013). Any factor influencing these processes favorably or adversely can impact growth and overall productivity. Furthermore, there is ample information about the protective role of different antioxidants in plant growth and development (Henmi et al., 2007).

Reactive oxygen species interacts with key-signaling components like calcium (Ca^2+^), kinases (CDPKs and MAPKs), cyclic nucleotides, G-proteins, several transcription factors (TFs), and other regulators (Devireddy et al., 2021). The intricate interaction between ROS and these signaling cues orchestrate a cardinal response involving an appropriate molecular, metabolic, and physiological acclimation responses, allowing the plant to survive under adverse environmental conditions (Zhu et al., 2016; Waszczak et al., 2018; Kollist et al., 2019; Zandalinas et al., 2020). The cell division and expansion are critical events of the plant growth process that are often affected by environmental stresses and are under the control of ROS, phytohormones, transcription factors, kinases, and small RNAs (Huang et al., 2019; Mishra et al., 2021b; Tiwari et al., 2021a,b, c). The role of ROS in cell proliferation is also evident from reports that reveal manganese superoxide dismutase (MnSOD) regulating a redox cycle within the cell cycle (Sarsour et al., 2014). Zhang et al. (2013) observed that *OsAPX2*-overexpressing plants were more tolerant to drought stress than wild-type plants at the booting stage, as revealed by a significant increase in spikelet fertility under abiotic stresses. ABA-independent pathways that regulate growth and development of rice (*Oryza sativa*) at both seedling and panicle emergence involve STRESS-RESPONSIVE NAC1 (SNAC1)-regulated downstream *PP2C* genes such as *OsPP18*, which confers drought and oxidative stress tolerance by regulating ROS homeostasis (You et al., 2014). Mitogen-activated protein kinase (MAPK) cascades mediate cell differentiation and development, hormonal activity, and abiotic stress responses (Komis et al., 2018). MAPK kinases have been characterized to impart stress resistance and ROS equilibrium in cotton (*Gossypium* sp.) (Lu et al., 2014) and pepper (*Capsicum* sp.) (Jeong et al., 2020). Overexpression of *GhMKK1* leads to significant accumulation of antioxidant enzymes and enhanced ROS scavenging activities, thereby improving tolerance of tobacco (*Nicotiana* sp.) plants to salt and drought stresses (Lu et al., 2014). During drought, the expression levels of MAPKs, *MdMAPK16-2*, *MdMAPK17*, and *MdMAPK20-1* were elevated compared to those in apple (*Malus domestica*) seedlings under control conditions (Huang et al., 2020). Huang et al. (2020) reported that arbuscular mycorrhizal fungi (AMF) could utilize the MAPKs signaling pathway to enhance drought tolerance by using MAPK signaling as an intermediate pathway for interactions between AMF and their host apple plant. Besides, the extensive role of MAPKs, other kinases such as histidine kinases, CDPKs, leucine-rich repeat-receptor-like kinases, and serine-threonine protein kinases are also at the center of stress tolerance (Mishra et al., 2021a; Tiwari et al., 2021a,b). Early leaf development is regulated by genes-encoding *OsSIK2*, a protein kinase located in rice leaf and sheath plasma membrane. The *OsSIK2* was suggested as a candidate gene for manipulation to facilitate crop improvement. It is hypothesized to integrate stress signals, predictably through ROS, into a developmental program for improved adaptation under stress conditions (Chen et al., 2013). Overexpression of calcium-dependent protein kinase gene, *OsCPK4*, results in increased tolerance to salt and drought stresses in rice plants. This was possibly due to the higher expression of several genes that regulate protection against oxidative stress and lipid metabolism, and thus determining cell membrane stability under stress conditions (Campo et al., 2014). The zinc finger super family transcription factors regulate multiple aspects of plant development and abiotic stress tolerance (Liu et al., 2021). Zinc Finger 2 (SlZF2) is rapidly induced by drought in cultivated tomatoes (*Solanum lycopersicum*), and its ectopic expression provides tolerance against salinity stress (Hichri et al., 2014). The chrysanthemum (*Chrysanthemum grandiflorum*) Zinc Finger Protein 1 (*CgZFP1*) shows enhanced expression under drought and salinity, and its heterologous expression in *Arabidopsis* revealed increased tolerance to both stresses.

The plant response to limited water availability varies with developmental growth stages of plants, which influence the final yield. There is ample evidence to support that ROS-AOS system influences on the cell division and expansion. Furthermore, many of the genes associated with growth and development of plants are linked to ROS-AOS. Some of the key genes include kinases such as MAPKs and those involved in Ca^2+^ signaling. Association of genes with ROS-AOS-mediated control of cell division and expansion could be genetically engineered appropriately to express or silence genes in response to sufficient or deficit soil moisture. Genetically engineered plants employ mechanisms in a way that cell division and expansion are continued or ceased based on water availability to ensure survival and productivity of plants. To counter mid-season dry spells, it would be appropriate to choose plants that have AOS-ROS system and associated traits tuned for survival till the next spell of water supply. Alternatively, exogenous application of ROS-scavenging biostimulants can be recommended as an effective management practice. A combination of the optimized AOS-ROS mechanism and external application of effective biostimulants have the potential to facilitate EUW by plants.

### Assimilate Synthesis

Cellular events associated with cell division and expansion are followed by the accumulation of assimilates during the growth process. Alteration in the photosynthetic process can affect plant growth (Weraduwage et al., 2016). Chloroplast is one of the major sites of ROS production, with photosynthesis affected directly due to the ROS-induced impact on photosynthetic apparatus and indirectly due to altered stomatal mechanisms. The ROS can cause protein breakdown, chlorophyll degradation, and lipid peroxidation in leaves, resulting in a loss of photosynthetic electron transport chain, membrane integrity, and cell death (Slooten et al., 1995; Ekmekci and Terzioglu, 2005). Higher levels of ROS are generated in plants when drought is accompanied with high solar irradiation and temperatures (Munne-Bosch et al., 2001; Jubany-Mari et al., 2010). Damage to cell membranes and reduction in CO_2_ intake due to closure of stomata restrict the capacity of plants to use photosynthetically active radiation (PAR) under soil moisture stress (Bota et al., 2004; Flexas et al., 2004; Chaves et al., 2009). This can be prevented by reducing the ROS generation and enhancing the detoxification of ROS (Das and Roychoudhury, 2014). The *OsCPK4* protects cellular membranes from stress-induced oxidative damage and thereby positively regulates salt and drought stress responses in rice (Campo et al., 2014). Previously, attempts have been made to improve the tolerance of plants under various abiotic stresses through protecting photosynthetic machinery by manipulating antioxidant enzymes, including the overexpression of SOD, GR, and dehydroascorbate reductase (DHAR) through genetic engineering (Logan et al., 2006). The strengthening of chloroplast antioxidant defenses could be the key protective mechanism for plant cells toward enhancing abiotic stress tolerance (Mishra et al., 2021b).

Drought-induced loss of chlorophyll accompanied by an elevated ratio of the violaxanthin pigment in chlorophyll effectively helps in mitigating oxidative load in the chloroplast by reduction of light-absorption centers (Brunetti et al., 2018). Carotenoids, particularly xanthophyll pigments, are substantially affected by drought stress, which play an effective role as singlet oxygen scavengers (Krieger-Liszkay, 2005). The ROS-detoxifying antioxidant enzymes and ascorbic acid are upregulated to protect membrane lipids from oxidation (Munne-Bosch et al., 2001; Du et al., 2010). Protection of photosynthetic system against excess H_2_O_2_ produced by photorespiration during drought was observed in tobacco due to overexpression of gene-coding ascorbate peroxidase (Yan et al., 2003). Mitochondrial Alternative Oxidase (AOX) has been hypothesized to aid photosynthetic metabolism by acting as an additional electron sink for the photogenerated reductant or by dampening ROS generation. This was essential for maintaining photosynthesis under mild drought in *Nicotiana tabacum* (Dahal et al., 2014).

Other metabolites, such as galactinol and raffinose, can function as osmoprotectants in plant cells. Transgenic *Arabidopsis* plants overexpressing galactinol synthases (*GolS1* and *GolS2*) exhibited increased accumulation of these oligosaccharides and increased tolerance to oxidative damage induced upon exposure to chilling and osmotic stress (Nishizawa et al., 2008). Biosynthesis of compatible solute glycinebetaine enhances the tolerance of plants to soil moisture stress (Chen and Murata, 2008). Glycinebetaine synthesis activates ROS-scavenging antioxidant enzymes and thus alleviates oxidative stress. Intriguingly, the elevated levels of glycinebetaine seem to be more effective in the chloroplasts compared to cytosol, indicating its competency to protect the photosynthetic machinery (Chen and Murata, 2008). Trehalose, a non-reducing disaccharide, accumulates to higher levels in some desiccation-tolerant plants like *Myrothamnus flabellifolius* and functions as an osmolyte and stabilizes proteins and membranes (Iordachescu and Imai, 2008).

Chloroplast acts as a site of photosynthesis as well as ROS production. Stress-mediated ROS generation impacts the membrane and also activates signaling related to stomatal closure. Therefore, the site of synthesis and accumulation of photosynthates are consistently associated with ROS-AOS system, which is evident from the literature cited in this section. Drought stress effect on chlorophyll is largely mitigated by accessory pigments, such as carotenoids and violaxanthin. They primarily scavenge ROS molecules to provide membrane stability and protection to maintain pigment system. The challenge is to design strategies at the molecular level to fine-tune best combinations of players for the light-trapping system to cease/decelerate their function when stomata tend to close for saving water. Although such a system might exist in nature, phenotyping methods to identify such genotypes may play a crucial role in improving the photosynthesis per unit available water, ultimately contributing to EUW.

### Assimilate Transport

Phloem transport plays an important role in maintaining homeostasis between plant growth and abiotic stress responses by providing an uninterrupted pathway of carbon distribution and signaling to all organs (Dinant and Lemoine, 2010; Ainsworth and Bush, 2011). Hydrostatic pressure gradient between the source and sink organs created during this process drives the mass flow of phloem sap. The phloem transport is maintained by a positive pressure energized and regulated by a dynamic loading between bundle sheath cells at source locations and the sieve element companion cell complex (Orians et al., 2015). The electrical signals involving Ca^2+^, Cl^–^, and K^+^ fluxes are transmitted rapidly along the length of the sieve element cell as well as laterally from parenchyma cells to sieve an element *via* companion cells through plasmodesmata (Borges, 2008) in response to stress and ultimately impact mobilization of carbohydrates.

Remobilization of pre-stored carbohydrates from wheat (*Triticum aestivum*) stems to grain results in a significant contribution to yield during terminal drought conditions (Bazargani et al., 2011). Studies conducted to underpin the molecular nexus responsible for stem reserve utilization during drought conditions in two contrasting wheat landraces (N49 and N14) revealed differential expressions of proteins with the highest levels in efficient landrace N49 at 20 days after anthesis. This is in conjunction with active remobilization of dry matter, indicating a probable involvement of these differential proteins in effective stem reserve remobilization in N49 (Bazargani et al., 2011). Furthermore, some proteins associated with this process were related to oxidative stress defense, suggesting ROS-AOS playing a role in remobilization of stem reserves (Bazargani et al., 2011).

Despite abundant stored reserves in stems, plants may fail to produce grains if environmental stresses affect reproductive organs. The maize transcriptome analysis involving well-watered and drought-treated fertilized ovary and basal leaf meristem tissue displayed a significantly decreased abundance of transcripts involved in the cell cycle and cell division only in the drought-stressed ovary (Kakumanu et al., 2012). Many of the genes were related to sucrose and starch metabolism changes in the ovary, consistent with a decrease in sucrose transporter function and starch levels. Possible mechanisms for adaptive responses included repression of a phospholipase C-mediated signaling pathway, activation of programmed cell death-mediated senescence, and arrest of the cell cycle in the stressed ovary a day after pollination (Kakumanu et al., 2012; Zhang et al., 2017). Furthermore, an elevated invertase level was observed in the stressed leaf meristem, resulting in maintenance of hexose levels at an “unstressed” level in that tissue, accompanied with lower ABA levels, providing drought resistance (Kakumanu et al., 2012). A possible role of ROS-AOS in this process cannot be ruled out as ROS are associated with programmed cell death. Other evidence that hints at the association of ROS-AOS emerges from studies on *AtSUC9*, a gene associated with sucrose transport in the cell. This gene gets induced by low sucrose levels and then mediates the balance of sucrose distribution and promotes ABA accumulation to enhance resistance to abiotic stresses in *Arabidopsis* (Jia et al., 2015). Similarly, the role of carbohydrate metabolism, carbohydrate profile, and sugar cleavage and transport determined pollen sterility in grain sorghum (*Sorghum bicolor*) exposed to heat stress (Jain et al., 2007). Increased membrane damage and enhanced ROS in pollen grains under heat stress result in loss of pollen viability in sorghum (Djanaguiraman et al., 2014). Increased ROS and decreased antioxidant enzymes in pollen and pistils were observed under heat stress in sorghum (Djanaguiraman et al., 2018a) and pearl millet (*Pennisetum glaucum*) (Djanaguiraman et al., 2018b). Impact of heat stress becomes more pronounced in the absence of sufficient soil moisture.

Assimilate transport through phloem directly determines the quantity and quality of yield. Drought stress affects cell division and cell cycle-related genes in addition to starch and sucrose metabolism. Sugar metabolism is required for cell growth, expansion, and division. Thus, ROS-AOS system plays a dual control on plant growth by regulating the cell cycle as well as sugar metabolism. Since there is a possible involvement of ROS-AOS both in assimilate transport from stems to reproductive parts and also in reproductive physiology, it can be hypothesized that these critical aspects of grain developmental process offer new dimension to develop strategies to manage ROS-AOS through genetic improvement or through exogenous application of promising formulations that can quench ROS. The dual regulation of starch metabolism and the cell cycle are very important in pollen or ovary development as these processes depend extensively on cell division as well as cell expansion. Higher EUW can be expected in genotypes identified based on their potential to manage ROS-AOS for facilitating better protection of pollen and ovary, in addition to supporting stem reserve mobilization during reduced soil moisture.

### Leaf Senescence

Photosynthesis can also be affected by leaf senescence induced by ROS, depending on the age of the leaf (Rosenwasser et al., 2013). Under limited or excess irradiation, coordinated biochemical, molecular, and physiological processes are required for photosynthetic apparatus acclimation and simultaneous achievement of optimal and effective photosynthetic functioning (Kreslavski et al., 2018). Due to these processes, leaves usually display a high ability to adjust to alteration of microclimate conditions. Leaf senescence is a genetically programmed process that leads to a decline in various cellular processes, including photosynthesis, and involves the remobilization of macromolecules, such as proteins and lipids (Tamary et al., 2019). It is mainly governed by the developmental age and is reprogrammed by environmental stresses, such as drought, salinity, heat, and other stresses. An overview of chloroplast protein degradation during leaf senescence and the roles of ROS has been reviewed earlier (Khanna-Chopra, 2012). Membrane proteins can be crucial as they are involved in leaf senescence through ROS (Jespersen et al., 2015). Genetic modification of genes such as *OsTZF1* could delay leaf senescence as this gene regulates genes associated with the ROS-AOS balance (Maruyama et al., 2013). Stress-induced leaf senescence under heat stress is associated with increased membrane damage, increased ROS, and reduced activity of antioxidant enzymes in soybean (Djanaguiraman and Prasad, 2010; Djanaguiraman et al., 2011) and sorghum (Djanaguiraman et al., 2014). While leaf senescence is an ultimate solution for reducing water loss through transpiration, other mechanisms keep the leaves functional under soil moisture stress. It is speculated that vacuolar phenylpropanoids may act as a secondary antioxidant system, which acts upon the depletion of primary antioxidant defenses to keep whole-cell H_2_O_2_ within sub-lethal concentration (Fini et al., 2012). The photoprotection mechanism against ROS was more conspicuous in younger than in mature leaves of common figs (*Ficus carica*) in response to a combination of high irradiation (∼1,300 μmolm^–2^ s^–1^) and increased temperature (∼35°C) at midday. Photo protective strategies in young leaves enabled them to minimize oxidative damage due to a competent antioxidative system relative to mature leaves (Mlinaric et al., 2016).

As leaves are the sites for photosynthesis as well as ROS production, maintaining a homeostasis during stress determines the survival of plants. There is sufficient evidence to support involvement of ROS in leaf senescence through programmed cell death and cell membrane damage, and hence altering physiological processes and functions in plants. The inherent strategy of plants to survive under drought involves reduction in transpiration through reduced leaf size or load on plants. When inevitable, systematic elimination of older leaves rather than young leaves can substantially contribute to reduction in consumption of water by plants, particularly during terminal drought. Therefore, genetic manipulation of genes for optimized ROS-AOS functionality and photosynthesis under water-limited conditions is much needed. The strategy should also involve simultaneous facilitation of stem reserve mobilization for grain development in crop plants.

## Conceptual Framework of Reactive Oxygen Species-Antioxidative System for Effective Use of Water

**FIGURE 1:**
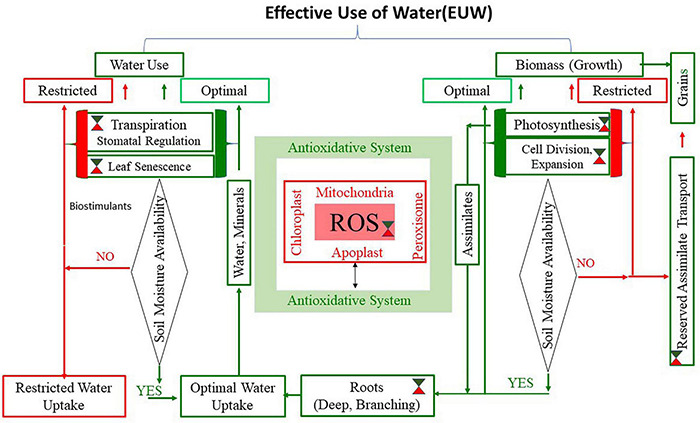
Water use by plants and biomass production are the two major components of Effective Use of Water (EUW). The figure is based on the fact that enhanced EUW is possible if water use by plants drives biomass production rather than biomass produced at a given time drives water use. The implications of reactive oxygen species and anti-oxidative system (ROS-AOS) that operates at mitochondria, chloroplast, peroxisome, and apoplast (shown at the center of the figure) are evident from scientific leads. The green/red symbol (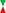 indicates opportunities to use ROS-AOS as feasible switches to put on or off the processes and functions required to achieve high EUW during optimal (green lines) and restricted (red lines) phases of soil moisture regime during the drought cycle. As shown in the Table 1, association of ROS-AOS is evident in the stomatal mechanism, water uptake and transport, and leaf senescence, which contribute to water use regulation while photosynthesis, cell division, and expansion assimilate transport, which are all linked with biomass production. Alternatively, exogenous bioactive compounds may be evaluated to get desired effect on each of the components to achieve maximum EUW based on the soil moisture regime, plant growth stage, and nature of drought.

We conceptualized that the strategy for enhancing EUW by the plant should prioritize water use-driven biomass production over biomass-driven water use under depleting or restricted soil moisture stress environments. As shown in Figure 1, the key components of this strategy should include a fine control over plant structure (shoots and roots), processes such as photosynthesis, cell expansion/proliferation, functions associated with water use, and biomass production. It is well-known that plants avoid water stress by extracting water through need-based extension and architecture of roots, often supported by tissue osmotic adjustment at root and shoot levels (Siddiqui et al., 2021; Strock et al., 2021). On the other hand, plants can save water by stomatal closure, reduced leaf expansion, leaf curling, and leaf rolling (Wang et al., 2020). Therefore, the EUW needs processes and structures that facilitate efficient usage of water that can contribute to survival during stress when soil moisture is deficit and maximum use for biomass production when sufficient water is available (Kosova et al., 2019). The conceptual framework encompasses major water use and growth processes, which is influenced by processes detailed in previous sections. The genes and endogenous substances addressed in this review are not exhaustive; instead, an attempt to reveal integrated ROS-AOS regulation governing adaptive mechanisms in plants exposed to drought has been presented. It needs to be critically examined if ROS-AOS facilitates selective switches for these processes and functions, depending on the type and growing habits of crop plants and the nature of drought stress. We discuss here the possible options for promoting water use-driven biomass production that underpins EUW of crop plants and hence improve productivity under drought stress conditions, such as early drought, mid-season or recurrent, and terminal drought.

Mid-season drought occurs when the depleting soil moisture is not replenished by natural precipitation and in the absence of irrigation. Since planting is carried out by the farmers only when there is optimum moisture in the soil, crop performance depends on the early establishment by utilizing available moisture (Fahad et al., 2017). Plants that develop their canopy rapidly have the advantage of enhancing the effective use of water by reducing moisture loss due to evaporation (Fahad et al., 2017; Sofi et al., 2019). Hence, ROS-mediated switches promoting biomass production processes and their distribution should be critical at the beginning of the soil moisture depletion curve (Laxa et al., 2019). The shoot growth has to be prioritized over roots for ensuring rapid ground cover at this phase to effectively utilize moisture at the top soil, followed by root proliferation to extract soil moisture from deeper layers of the soil profile. This preparedness for risk should be modulated by switches that impart endurance against limited soil moisture until the next opportunity emerges to replenish soil moisture. Under these conditions, cell division could continue, cell expansion could cease, but leaves continue to be functionally active. Plant growth can continue with partial closure of stomata, which facilitates carbon assimilation with minimum water loss (Zhang et al., 2018). This can happen if ROS-AOS switches are regulated to favor processes leading to increased growth under low vapor pressure deficit (VPD) periods within the diurnal cycle. This would induce a favorable response, as inactivation of chloroplast enzymes is known to occur under excess excitation energy, particularly during midday under tropical conditions (Foyer, 2018). Conditions during midday can lead to concomitant exposure to water stress, high sunlight irradiance, and high temperature, which significantly reduce the activity of enzymes aimed at detoxifying H_2_O_2_ (Sharma and Dubey, 2005; Guidi et al., 2008; Liu et al., 2011; Foyer, 2018; Aliyeva et al., 2020). Hence, an increase in violaxanthin pigments such as zeaxanthin from predawn to midday may avoid ROS generation by enhancing the capacity of thermal dissipation of excess radiant energy in the chloroplast (Kono and Terashima, 2014) and preserve thylakoid membranes from oxidation (Niinemets et al., 2003; Havaux et al., 2007). This may partially counter the depression of antioxidant enzymes activity.

Plants’ performance under intermittent drought is determined by water saving for survival and quick recovery after the cessation of drought stress. Roots can absorb water with less restriction early in the day, but the water demand would be greater than the supply during the latter part of the day (Neumann, 2008; Teran et al., 2019). With time, soil moisture is further depleted, and moisture stress tends to occur earlier and with higher intensity, affecting growth until replenishment of soil moisture. Thus, conservation of soil water by reduced transpiration would delay the onset of severe plant water-deficit stress and cessation of growth both diurnally and over a longer time period (Kulkarni et al., 2017). The concept of limited transpiration is based on plants modulation of water use through hydraulic conductivity during the early stages of crop development so that more water is available during the latter phase of the plant growth and development, which are more sensitive to stress conditions (Gholipoor et al., 2010, 2012). It has also been established that the effect of stomatal closure is less on carbon dioxide diffusion into the leaf than on water diffusion out of the leaf (Kono and Terashima, 2014; Qi et al., 2018). In this context, ROS switches need to be explored for promoting inherent or bio-regulator-induced water saving capacity at a critical stress period and efficient water uptake and utilization during favorable conditions. This is crucial for most dryland plants wherein the timing of water stress majorly determines the plant performance compared to the quantity of available water.

Terminal drought at the final stages of annual crops needs strategies that favor functional stay green with delayed senescence (Jagadish et al., 2015). Furthermore, under terminal drought, ROS-AOS is involved in the developmental shift from vegetative to the reproductive, particularly flowering and grain-filling stages (Jagadish et al., 2015). It is interesting to know that the control over genes (*SOC1* and *FUL*) associated with a shift from the vegetative to reproductive stage in *Arabidopsis* can significantly prolong the lifecycle of the plants, and, often, ROS-AOS is associated with these genes (Melzer et al., 2008; Lens and Melzer, 2012). Another gene, *Ghd7*, has been regarded as a critical regulator of heading date and yield potential in rice. This gene regulates yield traits through modulating panicle branching independent of the heading date. Drought is one among several factors that strongly repress the expression of *Ghd7* that maximizes the reproductive success of the rice plant (Weng et al., 2014). Furthermore, ROS-AOS switches need to be optimized to prevent damages to reproductive organs like pollens and ovules that may occur due to soil moisture stress (Kakumanu et al., 2012). In addition, plants could benefit from the action of ROS-AOS by promoting stem reserves for grain development and delayed senescence of roots for extracting soil moisture from deeper profiles of soil (Jagadish et al., 2015). This is crucial to maintain the normal physiological function of plants to support reproductive growth that can result in higher EUW. Possibilities of achieving this emerge from the fact that ROS-AOS are involved in the accumulation of compounds like oligosaccharides during the stress, which can be diverted for grain growth when photosynthesis ceases due to desiccation in plants (Cui et al., 2020; Ma et al., 2021). Annual crops are likely to benefit from opportunities with favorable periods in a diurnal cycle or intermittent precipitation during their short-life cycle. The physiological and molecular basis of ROS-AOS can help facilitate genetic improvement targeting traits for improved water relations in plants. Furthermore, the enhanced balance of ROS-AOS during critical growth stages can help tolerate drought episodes with minimal impact on biomass or economic yield (Aslam et al., 2015; Fang et al., 2015; Fang and Xiong, 2015; Salehi-Lisar and Bakhshayeshan-Agdam, 2016). The ROS-AOS facilitated acceleration or deceleration of processes associated with water uptake, and tissue water maintenance are crucial for optimum crop productivity under limited water conditions.

The proposed conceptual framework for translating ROS-AOS information for improvement in EUW is based on genetic improvement and traits identification that can alleviate stress. In addition to scientific leads that have emerged from physiological an molecular biology investigation on the role of ROS-AOS (Table 1) and access to evidence-based genetic variability is crucial for genetic improvement of tolerance of crop plants to abiotic stresses. Genetic variability in ROS production has been reported in grass species (Jespersen et al., 2015) and doubled haploid maize (Darko et al., 2009). Furthermore, genes identified through single-nucleotide polymorphism (SNP) and methylated quantitative trait locus (MQTL) approaches for genetic improvement of abiotic stress tolerance in wheat include those associated with scavenging of ROS and abscisic-acid-induced stomatal closure (Acuna-Galindo et al., 2015). Identified MQTL and candidate genes should be targeted for future studies and genetic improvement of abiotic stress tolerance. Recently, it has been observed that genes inhibiting anthocyanin synthesis at short high light exposure lose their action after prolonged exposure of plants to high light intensity, suggesting that these mechanisms might be facilitating acclimation to oxidative stress (Viola et al., 2016). Levels of antioxidants can be enhanced by silencing genes such as *OsSRFP*, which codes for stress-related RING Finger Protein 1 in rice (Fang et al., 2015; Fang and Xiong, 2015), and this can contribute to drought stress tolerance. Elevated antioxidant capacity may contribute to the paraquat tolerance of the paraquat-selected DH2 lines, and *in vitro* microspore selection represents a potential way to improve oxidative stress tolerance in maize (Darko et al., 2009).

**TABLE 1:** Some of the ROS-AOS genes associated with processes governing water use and biomass production.

S. No	Gene/TFs	Mode of action	Plant/Transformation receptor	References

**(A) Genes associated with water use and ROS-AOS: Stomatal closure**				
1.	Ca^2+^ ATPase gene (*OsACA6*)	Changes in several physiological indices	Tobacco	Huda et al. (2013)
2.	*OsCPK4* (Calcium dependent protein kinase)	ABA-induced antioxidant defense	Rice	Campo et al. (2014)
3.	*ZmCPK11* (Calcium dependent protein kinase)	Abscisic acid (ABA)-mediated stomatal movement	Maize	Ding et al. (2013)
4.	ZFP36 (abscisic acid (ABA)- and hydrogen peroxide (H_2_O_2_)-responsive C_2_H_2_-type ZFP gene)	ABA-induced upregulation of the expression and the activities antioxidant system Regulated by protein kinases (MAPKs) in ABA signaling (ABA-induced antioxidant defense)	Rice	Zhang et al. (2014)
5.	*OsTZF1* (CCCH-type zinc finger gene family)	Induced by abscisic acid, methyl jasmonate, salicylic acid and H_2_O_2_; Delays leaf senescence	Rice	Jan et al. (2013)
6.	Guard Cell Hydrogen Peroxide-Resistant 1 (GHR1)	ABA and hydrogen peroxide regulated stomatal movement under drought stress	*Arabidopsis* Soybean	Hua et al. (2012) and Tripathi et al. (2016)
7.	Cyclin H-I (CYCH-I)	Positively regulate stomatal opening by controlling ROS homeostasis	*Arabidopsis*	Zhou et al. (2014)
8.	Ring finger ubiquitin E3 ligase (*OsHTAS*)	Stomatal closure and ABA induced drought tolerance	Rice	Liu et al. (2016)
9.	Heme oxygenase (HY1)	ABA hypo/hyper sensitive regulation of stomatal closure	*Arabidopsis*	Xie et al. (2015)

**(B) Genes associated with water use and ROS-AOS: Root architecture**				

1.	NAC ANAC054/CUC1 (Downregulation)	Shoot apical meristem formation and auxin-mediated lateral root formation	*Arabidopsis*	Balazadeh et al. (2012)
2.	MADS AGL6/RSB1 (Downregulation)	Involved in axillary bud formation; control of flowering time and lateral organ development	*Arabidopsis*	Balazadeh et al. (2012)
3.	MYB MYB2	Inhibits cytokine-mediated branching	*Arabidopsis*	Balazadeh et al. (2012)
4.	Ca^2+^ ATPase gene (*OsACA6*)	Changes in several physiological indices	Tobacco	Huda et al. (2013)
5.	NAC2	Increase in root length	*Arabidopsis*	Gunapati et al. (2016)

**(C) Genes associated with water use and ROS-AOS: Leaf senescence**				

1.	MYB44	Regulates ethylene signaling	*Arabidopsis*	Balazadeh et al. (2012)
2.	NAC ANAC092/ORE1	Regulator of leaf senescence	*Arabidopsis*	Balazadeh et al. (2012)
3.	*GhTZF1* (cotton CCCH-type tandem zinc finger gene)	Delays leaf senescence by inhibiting reactive oxygen species accumulation	*Arabidopsis*	Zhou et al. (2014)
4.	MYB37/RAX1 (Downregulation)	Regulates axillary meristem formation	*Arabidopsis*	Balazadeh et al. (2012)
5.	*OsTZF1* (CCCH-type zinc finger gene family)	Induced by abscisic acid, methyl jasmonate, salicylic acid and H_2_O_2_; Delays leaf senescence	Rice	Jan et al. (2013)
6.	SNAC3 (ONAC003, LOC_Os01g09550)	Reduces levels of H_2_O_2_, malondialdehyde (MDA), and relative electrolyte leakage	Rice	Fang et al. (2015)
7.	Ca^2+^ ATPase gene (*OsACA6*)	Changes in several physiological indices	Tobacco	Huda et al. (2013)

**(D) Genes associated with growth and ROS-AOS: Photosynthesis**				

1.	NAC ANAC054/CUC1 (Downregulation)	Shoot apical meristem formation and auxin-mediated lateral root formation	*Arabidopsis*	Balazadeh et al. (2012)
2.	MYB37/RAX1 (Downregulation)	Regulates axillary meristem formation	*Arabidopsis*	Balazadeh et al. (2012)
3.	MYB44	Regulates ethylene signaling	*Arabidopsis*	Balazadeh et al. (2012)
4.	NAC ANAC092/ORE1	Regulator of leaf senescence	*Arabidopsis*	Balazadeh et al. (2012)
5.	*GhTZF1* (cotton CCCH-type tandem zinc finger gene)	Delays leaf senescence by inhibiting reactive oxygen species accumulation	*Arabidopsis*	Zhou et al. (2014)
6.	HB (HB2/HAT4)	Involved in cell expansion and cell proliferation	*Arabidopsis*	Balazadeh et al. (2012)
7.	(NAC) ANAC068	Mediates cytokine signaling during cell division	*Arabidopsis*	Balazadeh et al. (2012)
8.	(*PsAOX1* gene) Alternate oxidase gene	Regulated ROS by affecting alternate oxidase pathways	Pea	Dinakar et al. (2016)
9.	Ca^2+^ ATPase gene (*OsACA6*)	Changes in several physiological indices	Tobacco	Huda et al. (2013)
10.	*OsCPK4* (Calcium dependent protein kinase)	ABA-induced antioxidant defense	Rice	Campo et al. (2014)
11.	SNAC3 (ONAC003, LOC_Os01g09550)	Reduces levels of H_2_O_2_, malondialdehyde (MDA), and relative electrolyte leakage	Rice	Fang et al. (2015)

**(E) Genes associated with growth and ROS-AOS: Cell division and expansion**				

1.	Ca^2+^ATPase gene *(OsACA6*)	Changes in several physiological indices	Tobacco	Huda et al. (2013)
2.	MYB MYB37/RAX1 (Downregulation)	Regulates axillary meristem formation	*Arabidopsis*	Keller et al. (2006)
3.	NAC ANAC054/CUC1 (Downregulation)	Shoot apical meristem formation and auxin-mediated lateral root formation	*Arabidopsis*	Balazadeh et al. (2012)
4.	HB (HB2/HAT4)	Involved in cell expansion and cell proliferation	*Arabidopsis*	Balazadeh et al. (2012)
5.	(NAC) ANAC068	Mediates cytokinin signaling during cell division	*Arabidopsis*	Balazadeh et al. (2012)
6.	KUODA1 (KUA1)	Leaf development, decreases peroxidase activity	*Arabidopsis*	Lu et al. (2014)
7.	SNAC3 (ONAC003, LOC_Os01g09550)	Reduces levels of H_2_O_2_, malondialdehyde (MDA), and relative electrolyte leakage	Rice	Fang et al. (2015)
8.	*OsCPK4* (Calcium dependent protein kinase)	ABA-induced antioxidant defense	Rice	Campo et al. (2014)

**(F) Genes associated with growth and ROS-AOS: Shift from vegetative to reproductive phase**				

1.	MADS AGL6/RSB1 (Downregulation)	Involved in axillary bud formation; control of flowering lateral organ development	*Arabidopsis*	Balazadeh et al. (2012)
2.	MYB21	Petal and stamen development	*Arabidopsis*	Balazadeh et al. (2012)
3.	SEP2/AGL4 (Downregulation)	Flower and ovule development	*Arabidopsis*	Balazadeh et al. (2012)
4.	SEP1/AGL2 (Downregulation)	Flower and ovule development	*Arabidopsis*	Balazadeh et al. (2012)
5.	AP2/EREBP (RAV2/TEM2)	Repressor of flowering	*Arabidopsis*	Balazadeh et al. (2012)
6.	(NAC) ANAC089	Negative regulator of floral initiation	*Arabidopsis*	Balazadeh et al. (2012)
7.	AP2/EREBP (SNZ)	Represses flowering	*Arabidopsis*	Balazadeh et al. (2012)
8.	ABI3/VP1 AP2/B3-like	Seed development	*Arabidopsis*	Balazadeh et al. (2012)
9.	MADS SEP2/AGL4 (Downregulation)	Flower and ovule development	*Arabidopsis*	Balazadeh et al. (2012)
10.	MADS SEP1/AGL2 (Downregulation)	Involved in flower and ovule development	*Arabidopsis*	Balazadeh et al. (2012)
11.	WRKY51	Repression of jasmonate-mediated signaling	*Arabidopsis*	Balazadeh et al. (2012)
12.	WRKY25	Involved in response to various abiotic stresses	*Arabidopsis*	Balazadeh et al. (2012)
13.	SgNCED1	ABA-induced antioxidant defense (Drought and salt stress)	*Arabidopsis*	Zhang et al. (2009)
14.	*TaOPR1* (12-oxo-phytodienoic acid reductases)	ABA-induced antioxidant defense	*Arabidopsis*	Dong et al. (2013)
15.	Anther gene (Ghd7)	Regulates heading and yield potential	Rice	Weng et al. (2014)
				

Among the endogenous substances that can alleviate oxidative stress, polyamines such as putrescine reduce ROS activity in roots of red kidney bean (*Phaseolus vulgaris*) and alleviate Al-induced inhibition of root elongation. This was mediated by inhibition of NADPH oxidase activity (Wang et al., 2013), suggesting that deeper understanding of ROS-AOS switches can help in identifying useful bioregulators to achieve desired results in promoting EUW. Anthocyanins protect plant tissues against excess light and ROS by absorbing light and thus reducing the amount of energy that reaches the photosynthetic apparatus. Therefore, bio-regulators that promote anthocyanin production or protection can possess the potential to alleviate the soil moisture stress that accompanies exposure to high light (Hughes et al., 2013). Anthocyanins may also act as antioxidant compounds in plants since they are ROS scavengers (Nakabayashi et al., 2014).

Possibilities of employing ROS-AOS insights for management of drought are evident from a series of recent studies conducted with biostimulants in crops, including tomato (Casadesús et al., 2019; Antonucci et al., 2021, Francesca et al., 2021), capsicum (Agliassa et al., 2021), soybean (do Rosário Rosa et al., 2021), and lettuce (Zhou et al., 2022). Exogenous application of 16 tannin-based biostimulants having antioxidant properties improved root system architecture of tomato under salt stress (Campobenedetto et al., 2021). Additionally, *Late embryogenesis abundant protein (LEA) family proteins* were found to be upregulated, which are associated with water-limited stress tolerance. Thus, osmotic stress in crop plants can be managed, and this could be extended to managing drought stress. A commercial glycine-betaine-based biostimulant improved EUW in tomato under water stress condition. Moreover, elicitation of stress priming through induction of H_2_O_2_-mediated antioxidant mechanisms was suggested as one of the primary reasons behind the beneficial effect (Antonucci et al., 2021). Antioxidant properties of biostimulants are attributed to improvement in crop productivity under water stress (Malik et al., 2021). Studies reveal the potential of timely application of biostimulants (depending on the growth stage) either through foliar approaches or fertigation to improve drought tolerance. These recommendations can immensely help in improving effective use of water, as biostimulants can improve tolerance to water-deficit conditions and also facilitate recovery from drought stress.

## Way Forward

This review is central to contemporary demand for scientific interventions that can improve productivity of dryland crops, particularly when various environmental constraints challenge food security of millions of people. Dryland agriculture will play an increasingly important role in meeting food security challenges (Ng et al., 2012; Temam et al., 2019), mainly because of two reasons: First, only about 7% of the agricultural land across the world is equipped with irrigation (FAO et al., 2019), and the substantial remaining proportion of the area depends on rains and provides an opportunity to use technological advances to enhance productivity and support the most vulnerable populations; second, the world’s supply of freshwater for irrigation is insufficient to meet the targeted food production with current technology. Therefore, any technological interventions that can enhance the productivity of crops under limited water stress conditions will be critical to drylands that routinely encounter varying levels of drought stress (Rao and Gopinath, 2016; Rane and Minhas, 2017; Zuniga et al., 2021). Variability rather than the amount of total rainfall determines crop growth, development, and yield under dryland conditions. Therefore, we need location-specific drought-adaptive mechanisms or traits to develop crop varieties with improved resilience. In this context, adaptive mechanisms based on ROS-AOS are perceived to play important roles due to their involvement in almost all growth, development, and yield-determining processes throughout the crop growth cycle. Genotypes are known to differ in their response to drought stress, including the production of ROS and AOS mechanisms. Targeted traditional and molecular breeding programs using both conventional and novel genomic tools are essential to enhance drought-tolerant genotypes and hybrids in major food crops and address food and nutritional security around the world.

Knowing the ubiquitous and complex nature of ROS-AOS in plants (Noctor et al., 2014), it is important to advance scientific investigations in optimizing ROS-AOS under severe drought stress conditions to facilitate EUW in both annuals and perennial crops under dryland and rainfed environments. The majority of damage and losses in dryland are due to time and duration of occurrence rather than net soil moisture throughout the crop season. Therefore, fine-tuning of ROS-AOS mechanisms either genetically or through exogenous application of bio-regulators can help in optimizing the use of water for biomass production, crucial for maintaining productivity of crops. Such suggestions derive support from the evidence that the antioxidant machinery of plants can be more effective under mild than severe stress conditions (Guidi et al., 2011). A lot of promise emerges from the fact that several master regulators controlling the balance between growth and survival have been identified. Possibilities of association of ROS and antioxidant systems through these regulators have been predicted but are yet to be experimentally elucidated. If proven, this can open up new opportunities to promote plant growth and development without compromising on protective tolerance mechanisms (Claeys and Inze, 2013). Hence, it is necessary to explore the possibilities of exogenous bioregulators to ensure need-based neutralization of apoplastic-ROS that accumulate outside the cell membrane during drought. The need may be to reduce transpiration loss of water for survival of perennial fruits crops during prolonged drought or to keep the leaves of annuals photosynthetically and physiologically functional under mild stress. This will help promote rapid recovery after the stress is released or to provide sufficient time for assimilate transport to developing grains as stem reserve mobilization is crucial for grain growth (Bazargani et al., 2011; Jagadish et al., 2015). The role of optimized ROS-AOS in processes associated with the transport of assimilates in plants needs to be further investigated. Drought has been reported to either enhance or depress the activity of antioxidant enzymes, depending on species, genotypes, and stress severity (Ren et al., 2007; Liu et al., 2011; Oladosu et al., 2019). The issue of osmotic stress-induced changes in antioxidant enzymes activity and low molecular weight antioxidants, such as ascorbic acid, needs to be elucidated.

Novel findings from the leaf level under controlled environment have to be validated at the whole plant level in crops under field conditions as expression of transcripts depends on growth conditions as illustrated in case of inhibition of cell death-inducing genes such as LESION SIMULATING DISEASE 1 (LSD1) null mutant (lsd1), which are shown to improve tolerance to combined drought and high-light stress in *Arabidopsis* (Wituszynska et al., 2013). Furthermore, it is necessary to elucidate the role of such genes with those associated with apoptosis or phenoptosis, where the programmed death of cells, organs or organisms occurs (Skulachev and Skulachev, 2018). Furthermore, the control over genes such as *SOC1* and *FUL* associated with a shift from vegetative to the reproductive stage that prolongs the lifecycle of the plants through ROS-AOS needs attention. It is hypothesized that regulation of such genes may help accelerate or delay the leaf senescence based on the time of occurrence of the drought stress in the life cycle of plants. Despite the lack of significant links at the cellular level for the execution of several functions for appropriately manipulating the ROS-AOS, there are ample indications of its involvement in water use and growth (Table 1). This new knowledge and understanding provide the platform for an optimistic approach for effective scientific intervention for managing crops under dryland conditions through genetic improvement of crop adaptation through bioactive compounds, or a combination of both, for mitigating water-stress-induced loss in crop productivity.

## Author Contributions

JR, AS, SJ, and PP: conceptualization, review of literature, and preparation of manuscript. MT: review of literature. All authors contributed to the article and approved the submitted version.

## Conflict of Interest

The authors declare that the research was conducted in the absence of any commercial or financial relationships that could be construed as a potential conflict of interest.

## Publisher’s Note

All claims expressed in this article are solely those of the authors and do not necessarily represent those of their affiliated organizations, or those of the publisher, the editors and the reviewers. Any product that may be evaluated in this article, or claim that may be made by its manufacturer, is not guaranteed or endorsed by the publisher.
